# MicroRNAs in Methamphetamine-Induced Neurotoxicity and Addiction

**DOI:** 10.3389/fphar.2022.875666

**Published:** 2022-04-14

**Authors:** Bi Deng, Zhirui Zhang, Huixuan Zhou, Xinran Zhang, Shuliang Niu, Xisheng Yan, Jie Yan

**Affiliations:** ^1^ Department of Forensic Science, School of Basic Medical Science, Central South University, Changsha, China; ^2^ Xiangya School of Medicine, Central South University, Changsha, China; ^3^ School of Basic Medical Science, Xinjiang Medical University, Urumqi, China; ^4^ Department of Cardiovascular Medicine, Wuhan Third Hospital and Tongren Hospital of Wuhan University, Wuhan, China

**Keywords:** microRNAs, methamphetamine, neurotoxicity, addiction, treatment

## Abstract

Methamphetamine (METH) abuse remains a significant public health concern globally owing to its strong addictive properties. Prolonged abuse of the drug causes irreversible damage to the central nervous system. To date, no efficient pharmacological interventions are available, primarily due to the unclear mechanisms underlying METH action in the brain. Recently, microRNAs (miRNAs) have been identified to play critical roles in various cellular processes. The expression levels of some miRNAs are altered after METH administration, which may influence the transcription of target genes to regulate METH toxicity or addiction. This review summarizes the miRNAs in the context of METH use, discussing their role in the reward effect and neurotoxic sequelae. Better understanding of the molecular mechanisms involved in METH would be helpful for the development of new therapeutic strategies in reducing the harm of the drug.

## 1 Introduction

Methamphetamine (METH), also known as “ice,” is an amphetamine-type central nervous system (CNS) stimulant and a highly addictive psychostimulant (Monica et al., 2017). Given its easy synthesis and multiple ingestion pathways, METH is widely abused globally (Shaerzadeh et al., 2018). However, long-term use of METH inevitably causes damage to the CNS, resulting in psychosis, cognitive impairment, and neurodegenerative disease, which carries a severe global public health burden. Mounting evidence has been provided regarding METH action inside the body (Moratalla et al., 2017; Sambo et al., 2018; Chen et al., 2021a; Zhang et al., 2021). However, the cellular and molecular bases in response to the substance remain largely elusive. MicroRNAs (miRNAs) are eukaryotic non-coding RNA molecules about 19–25 nucleotides long (Lu and Rothenberg, 2018). They are believed to play a vital role in the morphology, neuronal development, and neuronal plasticity of the brain (Cao et al., 2016; Marangon et al., 2019). Many studies have found the function of miRNAs in regulating degenerative neural diseases, depression, schizophrenia, and drug addiction (Cao et al., 2016; Gu et al., 2020; Ma et al., 2020; Gowen et al., 2021). METH can induce the change of miRNA expression levels in several brain subregions, and some miRNAs have been shown to function in METH-related brain effects (Chavoshi et al., 2020; Sandau et al., 2020; Yang et al., 2020). In this review, we discuss the role of miRNA in METH-induced neurotoxicity and addiction, hoping to shed new light on the clinical application regarding its adverse consequences.

## 2 MiRNAs in the Neurotoxicity of METH

Neurotoxicity is defined as physical damage to the neurons. In a broader sense, it refers to the permanent or reversible adverse effects of substances on the structure and function of neurons, which results in the destruction of neuronal components and the anomaly of histological signs or behaviors (Moszczynska and Callan, 2017). METH is a highly neurotoxic substance and causes dopamine (DA) neuron damage through neuroinflammation (Dang et al., 2021), oxidative stress (McDonnell-Dowling and Kelly, 2017), hyperthermia (Matsumoto et al., 2014; Liao et al., 2021), and mitochondrial dysfunction (Shin et al., 2018). Despite the fact that important advances have been made in recent decades, the mechanism underlying the neurobiology of METH-induced neurotoxicity is not fully elucidated. The findings achieved so far have not led to the development of effective pharmacological treatments (Yang et al., 2018). In spite of the paucity of literature regarding miRNAs in METH-induced neurotoxicity, several studies have found that miRNAs participate in METH-related neuroinflammation (Bai et al., 2016; Yu et al., 2019). MiRNAs may also play a role in METH-induced oxidative stress and neuronal apoptosis (Du et al., 2016). Herein, we discuss the involvement of miRNAs in these processes. Although evidences that support a role for miRNAs in neurotoxicity are weak as compared with those in addiction, these early findings open a window for uncovering METH-induced toxic effects within the brain.

### 2.1 MiRNAs in Neuroinflammation

Reactive glial cell proliferation is considered a typical response to CNS damage and a sensitive marker of neuronal injury (Ares-Santos et al., 2013). The neuroinflammation, such as activation of microglia and release of proinflammatory molecules, has been extensively reported in METH-elicited neurotoxicity. METH-induced neuroinflammation mainly occur in dopaminergic related areas, including the striatum, substantia nigra pars compacta, etc. (Granado et al., 2011; Ares-Santos et al., 2012; Ares-Santos et al., 2013). The molecular mechanisms underlying the inflammatory processes mainly include activation of glial fibrillary acidic protein (GFAP), Nod-like Receptor Protein 3 (NLRP3), Toll-like receptors 4 (TLR4), and tumor necrosis factor (TNF) receptors, as well as release of interleukins (IL-1, IL-6) and TNF-α (Fernandes et al., 2016; Frank et al., 2016; Park et al., 2017).

Recently, in a neurotoxic mouse model, METH down-regulated the expression of miR-155-5p, which can bind to the 3′-UTR of Pellion1 (Peli1). Decrease of miR-155-5p facilitated the upregulation of Peli1 level and promoted the production and secretion of inflammatory factors (Yu et al., 2019). The evidence of miR-155-5p in modulating brain inflammation can also be found in chronic neuroinflammation studies, of which the level of miR-155-5p was positively correlated with the cytokines interferon (IFN)-γ, TNF-α, IL-1, and IL-6 (Cardoso et al., 2012; Butovsky et al., 2015; Al-Ghezi et al., 2019).

MiR-143 can regulate NLRP3 by targeting the p53-up-regulated modulator of apoptosis (PUMA) to affect microglia activation in METH exposure. The expression of miR-143 was down-regulated in BV2 cells after METH administration, which attenuated the inhibitory effect of miR-143 on PUMA expression, thereby activating NLRP3 inflammasomes and microglia (Zhang et al., 2016b; Du et al., 2019). In comparison, up-regulation of miR-143 was found in isolated human brain microvessels and several brain regions, such as the cortex, striatum, and midbrain. Increased miR-143 inhibited the expression of PUMA resulting in increased endothelial permeability and blood-brain barrier (BBB) damage after METH treatment (Bai et al., 2016). The inconsistent change trend of miR-143 may be due to the distinct models employed between studies. Therefore, we should consider the different responses of various cell types when studying the miRNAs in METH. Overall, these results offer novel evidence of miRNAs regarding METH-induced neuroinflammation. The strategy of pharmacotherapy based on the miR-143/PUMA axis may have translational potential.

### 2.2 MiRNAs in Oxidative Stress

Oxidative stress refers to the imbalance of reactive oxygen species (ROS) and endogenous antioxidants. Low concentrations of ROS are required in cell homeostasis, while excess ROS results in DNA damage, lipid peroxidation, and eventually cell death (van der Pol et al., 2019; Li et al., 2021a; Li et al., 2021b). METH leads to the oxidation of DA and produces a large number of ROS and lipid peroxides in the mitochondria, such as hydroxyl (OH), hydrogen peroxide (H_2_O_2_), and superoxide ions (O^2−^), resulting in mitochondrial dysfunction and irreversible cell damage (Yang et al., 2018; Kim et al., 2020). In fact, there is an interaction between miRNA and oxidative stress such that miRNA regulates oxidative stress, and oxidative stress contributes to the alteration of miRNA expression level (Engedal et al., 2018).

Nuclear factor erythroid 2-related factor-2 (Nrf2) is an endogenous antioxidant protein that maintains the balance of oxidation reactions (Kurinna and Werner, 2015), which has a substantial protective effect on METH-induced neurotoxicity (Ramkissoon and Wells, 2015; Meng et al., 2020). The lack of Nrf2 results in severe consequences *via* promoting oxidative stress and inflammation (Granado et al., 2011). There is no direct evidence that a specific miRNA regulates METH neurotoxicity through Nrf2. However, miR-181 and miR-495, which showed an up-regulation trend after METH administration, have been correlated with Nrf2 in other models (Sim et al., 2017). For example, miR-181 promoted chlorpyrifos-induced oxidative stress through Nrf2 and led to the occurrence and progress of Parkinson’s disease (Zhao et al., 2019). In the model of epileptic brain injury, upregulation of miR-495 negatively regulated Nrf2 to aggravate oxidative stress and neuronal apoptosis (Geng et al., 2018). Given the possible interaction between miRNA and Nrf2, miR-181, and miR-495 may regulate oxidative stress through Nrf2-related pathways in METH-induced brain insults (Kurinna and Werner, 2015; Ashrafizadeh et al., 2020). Further study was needed to clarify the role of these miRNAs in oxidative modulation.

### 2.3 MiRNAs in Neuronal Apoptosis

Specifically, METH triggers excessive DA release from synaptic terminals and affects the expression of multiple genes, which is of considerable significance in toxicity and relapse (Eskandarian Boroujeni et al., 2020; Fan et al., 2021). Excessive ROS generated from DA oxidation inhibits complexes I, II, III, and IV of the electron transport chain (ETC), resulting in mitochondrial dysfunction. Inhibition of ETC components enhances O_2_^−^ production due to electron leakage, which leads to disturbance of mitochondrial energy metabolism (Yang et al., 2018). Mitochondrial damage can thus result in a decrease in the expression of anti-apoptotic proteins B-cell lymphoma-2 (Bcl-2) and Bcl-xL, and an increase in pro-apoptotic proteins Bcl2-associated X protein (Bax) and p53 protein, which triggers the cell death cascade (Jayanthi et al., 2001; Yu et al., 2015). It is worth mentioning as well that METH not only kills DA terminals but neurons in the PFC and substantia nigra pars compacta (Ares-Santos et al., 2014; Tehrani et al., 2019). Studies have found that altered levels of miR-222, miR-24, and miR-195 are related to the p53 and mitogen-activated protein kinase (MAPK) signaling pathway in METH treatment. It is speculated that these miRNAs may regulate METH-dependent neuronal apoptosis (Du et al., 2016). MiR-222 and miR-24, which are upregulated after METH exposure, may directly bind to Bcl2 to inhibit its expression and promote neuronal apoptosis, and down-regulated miR-195 after METH administration may relieve the suppression of miR-195 to Bcl2, thereby attenuating neuronal apoptosis (Srivastava et al., 2011; Puerta-Gil et al., 2012; Singh and Saini, 2012; Du et al., 2016).

## 3 MiRNAs in METH Addiction

METH is known to transiently facilitate DA-mediated neurotransmission in specific brain circuits, notably the reward pathways. The canonical reward pathways extend from the ventral tegmental area (VTA) to the nucleus accumbens (Nac), dorsal striatum, hippocampus, and prefrontal cortex (PFC) through dopaminergic projections (Godino et al., 2015). Drug consumption in the addicted person is usually associated with an attenuated DA increase in these reward regions (Volkow et al., 2019; Chen et al., 2021a), which may be accompanied by a vicious circle of binge, sensitization, abstinence, and relapse (Volkow and Morales, 2015; Mizoguchi and Yamada, 2019). The essence of METH dependence and relapse is pathologically based on drug-induced gene expression and synaptic plasticity changes (Li et al., 2018; Chen et al., 2021b). In recent investigations, the expression level of miRNAs was associated with molecular events at the transcriptional and post-transcriptional levels in METH addiction.

### 3.1 METH-Induced miRNAs Changes in Brain Sub-Regions

#### 3.1.1 Nac

The Nac is one of the primary areas involved in regulating reward pathways. In a METH conditioned place preference (CPP) model, the levels of some miRNAs were highly changed, such as miR-124, miR-134, miR-496-3p, miR-194-5p, miR-200b-3p, miR-181a-5p, and miR-9. Because these miRNAs are mainly located in the synapto-dendritic compartment of synaptic plasticity and neuronal function, their expression changes may be closely related to METH addiction phenotypes (Sim et al., 2017).

The regulatory role of miR-124 has been reported in the study of cocaine. Chandrasekar et al. found down-regulated expression of miR-124 in the Nac after cocaine administration. The targets of miR-124 are comprised of brain-derived neurotrophic factor (BDNF), integrin β1, nucleus accumbens-associated protein 1 (NAC1), and axon-guidance molecules like semaphorin 6A (SEMA6A). These molecules were demonstrated conducive to the development of drug reward and addiction. Alteration of miR-124 level resulted in the change of BDNF expression to regulate the size of the spine, which was critical in cocaine-induced plastic reward and memory (Chandrasekar and Dreyer, 2009). Moreover, behavioral studies have demonstrated that overexpression of miR-124 in the Nac reduced cocaine-induced CPP, which can be reversed by down-regulated miR-124 after cocaine administration (Chandrasekar and Dreyer, 2011).

MiR-124 was also involved in argonaute2 (Ago2)-miRNAs-Dicer1 net, which may negatively influence METH addiction. Ago2 regulated miR-124 biogenesis through RNA-induced silencing complex (RISC) formation. When treated by METH, Ago2 was inhibited, resulting in the low expression of miR-124. Then the level of Dicer1 in the Nac was upregulated as the target of miR-124, which may be significant as the expression of Dicer1 in several brain regions is related to the response to abused drugs (Mulligan et al., 2013; Liu et al., 2019). Although the expression of miR-124 in Nac shares a similar descending trend between METH and cocaine, the underlying mechanisms may be distinct as the target genes vary between the investigations. More studies focusing on miR-124 would be of significance due to its activity across drug modalities. However, findings must be interpreted with caution because these studies did not determine miRNAs in the Nac core or shell. This is of critical importance because the functions between the two areas differ.

Repeated exposure to METH changes the cell morphology and synaptic function, which exhibits hyperresponsiveness to METH, termed psychomotor sensitization. In the METH sensitization model, some researchers have demonstrated that several miRNAs are significantly altered in Nac (Ni et al., 2019; Su et al., 2019; Li et al., 2021c; Liu et al., 2021). For example, decrease of miR-29c resulted in inhibition of DNA methyltransferase 3 (Dnmt3)a and Dnmt3b, which contributed to attenuation of METH-induced locomotor sensitization (Su et al., 2019). Besides, knockdown of miR-3068-5p, an Ago2-dependent miRNA, resulted in increased locomotor activity in mice, indicating a functional role of Ago2/miR-3068-5p cascade during the development of METH sensitization (Liu et al., 2021). In line with this finding, Liu et al. proposed that the downregulation of miRNAs in locomotor sensitization was likely owing to a reduction in Ago2-mediated splicing. Considering the essential role of Ago2 in miRNA biogenesis, we should realize that Ago2 may be an upstream regulator in METH sensitization, which needs further evidence to identify.

#### 3.1.2 Dorsolateral Striatum

The dorsolateral striatum plays an essential role in the transition from controlled or recreational drug use to addiction despite largely unknown mechanisms (Giuliano et al., 2019; Lipton et al., 2019). It reported that the expression of miR-134 in the dorsolateral striatum was upregulated after excessive or uncontrolled intake of METH (Shi et al., 2019). MiR-134 was a brain-specific miRNA regulating memory formation and synaptic plasticity (Gao et al., 2010). In a self-administration (SA) model, miR-134 inhibited the expression and activity of LIM kinase 1 (LIMK1) to increase METH intake. Decrease of miR-134 in the dorsolateral striatum reduced excessive drug intake and addictive behavior, confirming a role of miR-134 contributing to uncontrolled METH consumption (Shi et al., 2019). It is worth noting that the expression level of miR-134 in Nac is also upregulated. We know that initial or limited exposure to substances like METH, cocaine, and alcohol, will cause changes in the function of the Nac before spreading to the back area (Shi et al., 2019). Thus, miR-134 may play a role in the Nac and dorsolateral striatum. However, LIMK1 does not show any changes in Nac, implying that miR-134 may participate in METH action by other targets instead of LIMK1.

Other studies in this area have focused on miR-181a, a miRNA with decreased level in METH-induced CPP rats. MiR-181a has been shown to directly regulate the expression of γ-aminobutyric acid receptor subunit alpha 1 (GABAAα1) (Sengupta et al., 2013; Wang et al., 2021). However, in METH-addicted rats, both miR-181a and GABAAα1 are concurrently downregulated in the dorsal striatum. The authors claimed that endoplasmic reticulum-associated protein degradation (ERAD) mediated ubiquitin protein degradation of GABAAα1 (Jiao et al., 2017), and 36 target genes of miR-181a were cooperatively involved in endoplasmic reticulum-associated GABAAα1 protein degradation. So they suggested that miR-181a induced the ubiquitination of GABAAα1 by ERAD rather than directly participating in METH addiction (Wang et al., 2021).

#### 3.1.3 Hippocampus

The hippocampus remains a crucial site supporting learning and memory (Preston and Eichenbaum, 2013; Castilla-Ortega et al., 2016). It plays a vital role in regulating motor activity, exercise capacity, and the spatial memory of METH addiction (Yan et al., 2019). Changes to miRNAs in the hippocampus were revealed in METH-induced addiction phenotypes. For example, the expression of miR-31-3p was upregulated after METH administration. MiR-31-3p inhibited the expression of Ras Homolog Family Member A (RhoA), a small GTPase, to regulate neuronal morphology and synaptic plasticity, enhancing METH-induced CPP in mice (Qian et al., 2021). To our knowledge, miR-31-3p was mainly identified as a biomarker or regulator in tumor research (Hawryluck and Brindley, 2018; Oshima et al., 2021), whereas rarely reported in neurological disorders. Moreover, in a CPP model, miR-183-5p was significantly upregulated in the hippocampus. The expression level of neuregulin-1 (NRG1) was down-regulated as a target of miR-183-5p, which is an indicator of METH dependence (Itzhak et al., 2015; Zhou et al., 2021). These results provide initial evidence that miR-31-3p and miR-183-5p may exert their function in the hippocampus to develop METH addiction. However, CPP is an experimental and controlled animal model; hence, verifying the results by using the self-administration (SA) model would be of great significance as it can better mimic METH ingestion in abusers.

#### 3.1.4 The VTA and PFC

VTA is the primary source of DA to the Nac and hippocampus (Hyman et al., 2006; Volkow et al., 2019). Microarray analysis of miRNAs in the VTA region after METH administration showed that the expression of 78 miRNAs was altered, and only seven miRNAs showed up-regulation. In this study, miR-30b-5p was significantly downregulated (Bosch et al., 2015). Although it is not sure whether miR-30b-5p participates in METH addiction, it has the most connection with target genes following alcohol and nicotine-alcohol exposure (Kazemi et al., 2020). In a study of the genetic changes of nicotine in the VTA, miR-30b-5p had a significant probability of participating in DAergic synaptic pathways, and the enrichment of this pathway supported the hypothesis that the mesocorticolimbic DA pathway was involved in the reinforcing effects of nicotine abuse (Keller et al., 2018). Given that METH shares a similar mechanism with nicotine regarding DA release, the role of miR-30b-5p in reinforcement and neuroplasticity of METH-abused models is worth exploring.

The microsequence analysis of miRNAs in response to METH was also studied in the PFC. The authors found 28 miRNAs differentially expressed dose-dependently in the SA model. After a low dose of METH administration, the expression of miR-195 was down-regulated, while the expression of miR-24 and miR-127 was upregulated with increased drug doses (Du et al., 2016). The functions of the three miRNAs are related to neurotrophic signaling pathways and axon guidance. miR-195 is one of the post-transcriptional inhibitors of BDNF. In the low-dose group, down-regulated miR-195 increased BDNF protein expression to regulate synaptic plasticity and maintain METH intake (Mellios et al., 2008). In comparison, the up-regulation of miR-24 and miR-127 after high-dose METH exposure may reduce the LIMK2 activity, so as to change the morphology of dendritic spines and affect drug addiction (Meng et al., 2003; Zhou et al., 2014; Du et al., 2016). The differential expression of miRNAs under low- and high-dose of METH may indicate different regulatory mechanisms in PFC. Nonetheless, it should be borne in mind that diverse factors, such as different technological platforms of sequencing, distinct criteria for determining miRNA up-or down-regulation, region-dependent repositories in the brain, and different postmortem intervals of sample materials might all introduce bias into the data. All of these factors should be considered when evaluating the role of the candidate miRNAs proposed by informatics.

## 4 Conclusion and Perspectives

MiRNAs have great potential to target genes and gene products currently unavailable for drugs (Nunomura and Perry, 2020). The small size of miRNA is convenient for synthesis and manipulation (Cao et al., 2016). Moreover, miRNAs are considered attractive therapeutic tools for the low toxicity of their endogenous expression (Braoudaki and Lambrou, 2015). Both miRNA-mimics and antagomirs are used to regulate intracellular miRNA (Leggio et al., 2017). These facilitate the application of miRNAs as a new therapeutic target and biomarker in many diseases (Leggio et al., 2017; Saliminejad et al., 2019). MiRNAs are widely enriched in the CNS to regulate the growth and development of neurons. They play a key role in the pathogenesis of neurodegeneration, including Alzheimer’s disease, Parkinson’s disease, ataxia, and drug-induced neurotoxicity (Cao et al., 2016) (Table 1). In drug addiction, miRNAs regulate synaptic plasticity through interactions with transcription factors to generate more persistent neuroplastic changes at the cellular level (Li and van der Vaart, 2011; Gowen et al., 2021) (Table 2).

**TABLE 1:** MiRNAs in METH-induced neurotoxicity.

MicroRNAs	Drugs	Species	METH dosing regimen	MicroRNA alteration	Downstream molecules	References
miR-143	METH	C57BL/6N mice	escalated doses of 1.5, 4.5, 7.5, 10 mg/kg i.p. for 8 days	Up	PUMA	Bai et al. (2016)
miR-143	METH	C57BL/6 J mice	30 mg/kg i.p. ×4, at 2 h intervals	Down	PUMA, NLRP3	Du et al. (2019)
miR-155-5p	METH	C57BL/6J mice	10 mg/kg i.p. twice daily for 7 days	Down	Peli1	Yu et al. (2019)
miR-181	METH	Wistar rats	escalated doses of 0.25, 0.5, 1, 2, 3, 4, 5 mg/kg i.p. for 15 days	Up	Nrf2?	Sim et al. (2017), Zhao et al. (2019)
miR-495	METH	Wistar rats	escalated doses of 0.25, 0.5, 1, 2, 3, 4, 5 mg/kg i.p. for 15 days	Up	Nrf2?	Sim et al. (2017), Geng et al. (2018)
miR-124	cocaine	BV-2 cells, rPMs	10 µM for 6, 12, 24 h	Down	MyD 88, IRAK 1, TRAF 6, KLF 4, TLR 4	Periyasamy et al. (2018)
miR-138	morphine	C57BL/6N mice	10 mg/kg, i.p. three times daily, with an increment of 5 mg/kg for 6 days	Up	TLR 7	Liao et al. (2020)

**TABLE 2:** MiRNAs in METH addiction.

Brain sub-regions	MicroRNAs	Drugs	Species	METH dosing regimen	MicroRNA alteration	Downstrea-m molecules	References
Nac	miR-124	METH	C57BL/6 mice	2 mg/kg i.p. for 7 days, abstinence 2 days	Down	Dicerl	Liu et al. (2019)
		Cocaine	Wistar rats	15 mg/kg i.p. for 15 days	Down	BDNF, integrin β1, NAC1, SEMA6A	Chandrasekar and Dreyer (2009)
	miR-128	METH	C57BL/6 J mice	2 mg/kg i.p. for 5 days, injection-free for 2 days, 2 mg/kg i.p. on 8th day	Up	Arf6, Cpeb3,Nlgn1	Li et al. (2021c)
	miR-29c	METH	C57BL/6 mice	0.9% saline 10 ml/kg ip. for 2 days, 2 mg/kg i.p. for 5 days, injection-free for 2 days, 2 mg/kg i.p. on 10th day	Down	Dnmt3a, Dnmt3b	Su et al. (2019)
	miR-204-3p	METH	C57BL/6 mice	0.9% saline 10 ml/kg i.p. for 2 days,2 mg/kg i.p. for 5 days, injection-free for 2 days, 2 mg/kg i.p. on 10th day	Up	Sema3A, Plxna4	Ni et al. (2019)
	miR-3068-5p	METH	C57BL/6 J mice	0.9% saline ip. for 2 days, 2 mg/kg i.p. for 5 days, injection-free for 2 days,2 mg/kg i.p. on 10th day	Down	Grin1	(Liu et al., 2021)
	miR-9-5p	METH	Wistar rats	continuous alternated doses of 0.25, 0.5, 1, 2, 3, 4, 5 mg/kg i.p. for 15 days	Up	BDNF	Sim et al. (2017)
VTA	miR-30b	METH	SD rats	0.1 mg/kg infusion in SA	Down	? unknown	Bosch et al. (2015)
		nicotine and/or alcohol	SD rats	6 mg/kg nicotine, blood alcohol concentrations between 80 and 180 mg/dl, for 4 w	Down	GNAI2, COTL1	Kazemi et al. (2020)
	miR-145	METH	SD rats	0.1 mg/kg infusion in SA	Down	HDAC2	Bosch et al. (2015)
	miR-129	METH	SD rats	0.1 mg/kg infusion in SA	Down	HDAC2	Bosch et al. (2015)
	miR-29	METH	SD rats	0.1 mg/kg infusion in SA	Down	HDAC4	Bosch et al. (2015)
PFC	miR-195	METH	SD rats	0.05 mg/kg infusion in SA for 12–13 days, 1 h daily for 14 days	Down	BDNF	Du et al. (2016)
	miR-24	METH	SD rats	0.05 mg/kg infusion in SA for 12–13 days, 6 h daily for 14 days	Up	LIMK2	Du et al. (2016)
	miR-127	METH	SD rats	0.05 mg/kg infusion in SA for 12–13 days, 6 h daily for 14 days	Up	LIMK2	Du et al. (2016)
Dorsolateral striatum	miR-134	METH	SD rats	0.05 mg/kg infusion in SA 2 h for 5 days, 6 h daily for 13–15 days	Up	LIMK1	Shi et al. (2019)
	miR-181a	METH	SD rats	1 mg/kg i.p. for 1 day × 4, 1 mg/kg saline for 1 day × 4 within 8 days	Down	GABAAα1	Wang et al. (2021)
Hippocampus	miR-31-3p	METH	C57BL/6J mice	1 mg/kg i.p. ×4 within 8 days	Up	RhoA	Qian et al. (2021)
	miR-183-5p	METH	Kunming mice	2 mg/kg s.c., 0.9% physiological saline, 10 ml/kg, s.c. for 6 days	Up	NRG1	Zhou et al. (2021)

METH, methamphetamine; i.p., intraperitoneal injections; i.v., intravenous; s.c., subcutaneous injection; SA, self-administration; SD, sprague-dawley; Nac, nucleus accumbens; VTA, ventral tegmental area; PFC, prefrontal cortex; rPMs, rat primary microglial cells.

The studies so far provide a rationale that miRNA can be utilized as a therapeutic tool in METH abuse. However, there are some limitations to be addressed. For years, the delivery of miRNA to the brain mainly relies on recombinant adeno-associated viruses. Now, it has been found that non-viral vectors may significantly increase the delivery efficiency of miRNAs in the brain (Zhang et al., 2013; Lee et al., 2019). Additionally, it is necessary to consider the miRNA crossing BBB to reach the specific parts of the brain and avoid activation of immune cells to produce toxicity (Almutairi et al., 2016; Leggio et al., 2017; Goh et al., 2019). In general, miRNAs are delivered across BBB by exosomes, nanoparticles, and the carrier system which binds to nicotinic acetylcholine receptors (Lee et al., 2019; Xia et al., 2019). However, whether miRNAs that cross the BBB are affected by METH-induced BBB damage is unknown. Further, as mentioned above, miRNAs play a role in multiple regions of the brain with distinct mechanisms to inhibit the expression of all the targeted genes at the same time. This may lead to contradictory effects in developing addiction, as well as protection in one area while promoting damage in another. Moreover, the inaccurate targeted delivery results from miRNAs binding to multiple target genes will cause unnecessary side effects and toxicity, also called off-target effects (Braoudaki and Lambrou, 2015; Cao et al., 2016).

It is sometimes difficult to determine whether a specific miRNA contributes to neurotoxicity or addiction. Generally speaking, miRNAs involved in the model of SA, CPP and locomotor sensitization are considered related to addiction. The establishment of the model was based on repeated-intermittent low dosing treatment of METH (no more than 2 mg/kg/day). By contrast, miRNAs may function in oxidative stress, apoptosis, and neuroinflammation models of METH, in which a relatively high dose of METH (over 2 mg/kg/day *in vivo*) was used. Besides, *in vitro* studies are commonly used to evaluate the toxicity of METH. It seems arbitrary for the classification just according to the criterion of dose regimen. Even at the low dose, METH may generate toxic effects in rodent brains, and drug users.

Overall, the study of miRNA in this field is still in its infancy. Although large bodies of data concerning METH-related miRNAs have been generated in recent years, the relationship between miRNA and METH is largely obscure. Translation of these observations into clinical practice is facing enormous challenges. More aggressive efforts remain to be made before reaching a clear understanding of miRNAs in METH action.

## References

[B1] Al-Ghezi Z. Z. Miranda K. Nagarkatti M. Nagarkatti P. S. (2019). Combination of Cannabinoids, Δ9- Tetrahydrocannabinol and Cannabidiol, Ameliorates Experimental Multiple Sclerosis by Suppressing Neuroinflammation through Regulation of miRNA-Mediated Signaling Pathways. Front. Immunol. 10, 1921. 10.3389/fimmu.2019.01921 31497013 PMC6712515

[B2] Almutairi M. M. Gong C. Xu Y. G. Chang Y. Shi H. (2016). Factors Controlling Permeability of the Blood-Brain Barrier. Cell Mol Life Sci 73 (1), 57–77. 10.1007/s00018-015-2050-8 26403789 PMC11108286

[B3] Ares-Santos S. Granado N. Espadas I. Martinez-Murillo R. Moratalla R. (2014). Methamphetamine Causes Degeneration of Dopamine Cell Bodies and Terminals of the Nigrostriatal Pathway Evidenced by Silver Staining. Neuropsychopharmacology 39 (5), 1066–1080. 10.1038/npp.2013.307 24169803 PMC3957101

[B4] Ares-Santos S. Granado N. Moratalla R. (2013). The Role of Dopamine Receptors in the Neurotoxicity of Methamphetamine. J. Intern. Med. 273 (5), 437–453. 10.1111/joim.12049 23600399

[B5] Ashrafizadeh M. Ahmadi Z. Samarghandian S. Mohammadinejad R. Yaribeygi H. Sathyapalan T. (2020). MicroRNA-mediated Regulation of Nrf2 Signaling Pathway: Implications in Disease Therapy and protection against Oxidative Stress. Life Sci. 244, 117329. 10.1016/j.lfs.2020.117329 31954747

[B6] Bai Y. Zhang Y. Hua J. Yang X. Zhang X. Duan M. (2016). Silencing microRNA-143 Protects the Integrity of the Blood-Brain Barrier: Implications for Methamphetamine Abuse. Sci. Rep. 6, 35642. 10.1038/srep35642 27767041 PMC5073292

[B7] Bosch P. J. Benton M. C. Macartney-Coxson D. Kivell B. M. (2015). mRNA and microRNA Analysis Reveals Modulation of Biochemical Pathways Related to Addiction in the Ventral Tegmental Area of Methamphetamine Self-Administering Rats. BMC Neurosci. 16, 43. 10.1186/s12868-015-0186-y 26188473 PMC4506769

[B8] Braoudaki M. Lambrou G. I. (2015). MicroRNAs in Pediatric central Nervous System Embryonal Neoplasms: the Known Unknown. J. Hematol. Oncol. 8, 6. 10.1186/s13045-014-0101-5 25652781 PMC4333163

[B9] Butovsky O. Jedrychowski M. P. Cialic R. Krasemann S. Murugaiyan G. Fanek Z. (2015). Targeting miR-155 Restores Abnormal Microglia and Attenuates Disease in SOD1 Mice. Ann. Neurol. 77 (1), 75–99. 10.1002/ana.24304 25381879 PMC4432483

[B10] Cao D. D. Li L. Chan W. Y. (2016). MicroRNAs: Key Regulators in the Central Nervous System and Their Implication in Neurological Diseases. Int. J. Mol. Sci. 17 (6). 842. 10.3390/ijms17060842 PMC4926376 27240359

[B11] Cardoso A. L. Guedes J. R. Pereira de Almeida L. Pedroso de Lima M. C. (2012). miR-155 Modulates Microglia-Mediated Immune Response by Down-Regulating SOCS-1 and Promoting Cytokine and Nitric Oxide Production. Immunology 135 (1), 73–88. 10.1111/j.1365-2567.2011.03514.x 22043967 PMC3246654

[B12] Castilla-Ortega E. Serrano A. Blanco E. Araos P. Suárez J. Pavón F. J. (2016). A Place for the hippocampus in the Cocaine Addiction Circuit: Potential Roles for Adult Hippocampal Neurogenesis. Neurosci. Biobehav Rev. 66, 15–32. 10.1016/j.neubiorev.2016.03.030 27118134

[B13] Chandrasekar V. Dreyer J. L. (2009). microRNAs miR-124, Let-7d and miR-181a Regulate Cocaine-Induced Plasticity. Mol. Cel Neurosci 42 (4), 350–362. 10.1016/j.mcn.2009.08.009 19703567

[B14] Chandrasekar V. Dreyer J. L. (2011). Regulation of MiR-124, Let-7d, and MiR-181a in the Accumbens Affects the Expression, Extinction, and Reinstatement of Cocaine-Induced Conditioned Place Preference. Neuropsychopharmacology 36 (6), 1149–1164. 10.1038/npp.2010.250 21307844 PMC3079833

[B15] Chavoshi H. Boroujeni M. E. Abdollahifar M. A. Amini A. Tehrani A. M. Moghaddam M. H. (2020). From Dysregulated microRNAs to Structural Alterations in the Striatal Region of METH-Injected Rats. J. Chem. Neuroanat. 109, 101854. 10.1016/j.jchemneu.2020.101854 32795519

[B16] Chen L. Huang S. Yang C. Wu F. Zheng Q. Yan H. (2021a). Blockade of β-Adrenergic Receptors by Propranolol Disrupts Reconsolidation of Drug Memory and Attenuates Heroin Seeking. Front. Pharmacol. 12, 686845. 10.3389/fphar.2021.686845 34113256 PMC8185332

[B17] Chen L. Yan H. Wang Y. He Z. Leng Q. Huang S. (2021b). The Mechanisms and Boundary Conditions of Drug Memory Reconsolidation. Front. Neurosci. 15, 717956. 10.3389/fnins.2021.717956 34421529 PMC8377231

[B18] Dang J. Tiwari S. K. Agrawal K. Hui H. Qin Y. Rana T. M. (2021). Glial Cell Diversity and Methamphetamine-Induced Neuroinflammation in Human Cerebral Organoids. Mol. Psychiatry 26 (4), 1194–1207. 10.1038/s41380-020-0676-x 32051547 PMC7423603

[B19] Du H. Y. Cao D. N. Chen Y. Wang L. Wu N. Li J. (2016). Alterations of Prefrontal Cortical microRNAs in Methamphetamine Self-Administering Rats: From Controlled Drug Intake to Escalated Drug Intake. Neurosci. Lett. 611, 21–27. 10.1016/j.neulet.2015.11.016 26592480

[B20] Du L. Shen K. Bai Y. Chao J. Hu G. Zhang Y. (2019). Involvement of NLRP3 Inflammasome in Methamphetamine-Induced Microglial Activation through miR-143/PUMA axis. Toxicol. Lett. 301, 53–63. 10.1016/j.toxlet.2018.10.020 30394308

[B21] Engedal N. Žerovnik E. Rudov A. Galli F. Olivieri F. Procopio A. D. (2018). From Oxidative Stress Damage to Pathways, Networks, and Autophagy via MicroRNAs. Oxid Med. Cel Longev 2018, 4968321. 10.1155/2018/4968321 PMC5932428 29849898

[B22] Eskandarian Boroujeni M. Peirouvi T. Shaerzadeh F. Ahmadiani A. Abdollahifar M. A. Aliaghaei A. (2020). Differential Gene Expression and Stereological Analyses of the Cerebellum Following Methamphetamine Exposure. Addict. Biol. 25 (1), e12707. 10.1111/adb.12707 30714656

[B23] Fan E. Xu Z. Yan J. Wang F. Sun S. Zhang Y. (2021). Acute Exposure to N-Ethylpentylone Induces Developmental Toxicity and Dopaminergic Receptor-Regulated Aberrances in Zebrafish Larvae. Toxicol. Appl. Pharmacol. 417, 115477. 10.1016/j.taap.2021.115477 33667508

[B24] Fernandes N. C. Sriram U. Gofman L. Cenna J. M. Ramirez S. H. Potula R. (2016). Methamphetamine Alters Microglial Immune Function through P2X7R Signaling. J. Neuroinflammation 13 (1), 91. 10.1186/s12974-016-0553-3 27117066 PMC4847215

[B25] Frank M. G. Adhikary S. Sobesky J. L. Weber M. D. Watkins L. R. Maier S. F. (2016). The Danger-Associated Molecular Pattern HMGB1 Mediates the Neuroinflammatory Effects of Methamphetamine. Brain Behav. Immun. 51, 99–108. 10.1016/j.bbi.2015.08.001 26254235 PMC5652313

[B26] Gao J. Wang W. Y. Mao Y. W. Gräff J. Guan J. S. Pan L. (2010). A Novel Pathway Regulates Memory and Plasticity via SIRT1 and miR-134. Nature 466 (7310), 1105–1109. 10.1038/nature09271 20622856 PMC2928875

[B27] Geng J. F. Liu X. Zhao H. B. Fan W. F. Geng J. J. Liu X. Z. (2018). LncRNA UCA1 Inhibits Epilepsy and Seizure-Induced Brain Injury by Regulating miR-495/Nrf2-ARE Signal Pathway. Int. J. Biochem. Cel Biol 99, 133–139. 10.1016/j.biocel.2018.03.021 29608952

[B28] Giuliano C. Belin D. Everitt B. J. (2019). Compulsive Alcohol Seeking Results from a Failure to Disengage Dorsolateral Striatal Control over Behavior. J. Neurosci. 39 (9), 1744–1754. 10.1523/JNEUROSCI.2615-18.2018 30617206 PMC6391574

[B29] Godino A. Jayanthi S. Cadet J. L. (2015). Epigenetic Landscape of Amphetamine and Methamphetamine Addiction in Rodents. Epigenetics 10 (7), 574–580. 10.1080/15592294.2015.1055441 26023847 PMC4622560

[B30] Goh S. Y. Chao Y. X. Dheen S. T. Tan E. K. Tay S. S. (2019). Role of MicroRNAs in Parkinson's Disease. Int. J. Mol. Sci. 20 (22). 10.3390/ijms20225649 PMC6888719 31718095

[B31] Gowen A. M. Odegaard K. E. Hernandez J. Chand S. Koul S. Pendyala G. (2021). Role of microRNAs in the Pathophysiology of Addiction. Wiley Interdiscip. Rev. RNA 12 (3), e1637. 10.1002/wrna.1637 33336550 PMC8026578

[B32] Granado N. Lastres-Becker I. Ares-Santos S. Oliva I. Martin E. Cuadrado A. (2011). Nrf2 Deficiency Potentiates Methamphetamine-Induced Dopaminergic Axonal Damage and Gliosis in the Striatum. Glia 59 (12), 1850–1863. 10.1002/glia.21229 21882243

[B33] Gu W. J. Zhang C. Zhong Y. Luo J. Zhang C. Y. Zhang C. (2020). Altered Serum microRNA Expression Profile in Subjects with Heroin and Methamphetamine Use Disorder. Biomed. Pharmacother. 125, 109918. 10.1016/j.biopha.2020.109918 32036213

[B34] Hawryluck L. Brindley P. G. (2018). Psychological Burnout and Critical Care Medicine: Big Threat, Big Opportunity. Intensive Care Med. 44 (12), 2239–2241. 10.1007/s00134-018-5063-6 29353460

[B35] Hyman S. E. Malenka R. C. Nestler E. J. (2006). Neural Mechanisms of Addiction: the Role of Reward-Related Learning and Memory. Annu. Rev. Neurosci. 29, 565–598. 10.1146/annurev.neuro.29.051605.113009 16776597

[B36] Itzhak Y. Ergui I. Young J. I. (2015). Long-term Parental Methamphetamine Exposure of Mice Influences Behavior and Hippocampal DNA Methylation of the Offspring. Mol. Psychiatry 20 (2), 232–239. 10.1038/mp.2014.7 24535458

[B37] Jayanthi S. Deng X. Bordelon M. McCoy M. T. Cadet J. L. (2001). Methamphetamine Causes Differential Regulation of Pro-death and Anti-death Bcl-2 Genes in the Mouse Neocortex. FASEB J. 15 (10), 1745–1752. 10.1096/fj.01-0025com 11481222

[B38] Jiao D. L. Chen Y. Liu Y. Ju Y. Y. Long J. D. Du J. (2017). SYVN1, an ERAD E3 Ubiquitin Ligase, Is Involved in GABAAα1 Degradation Associated with Methamphetamine-Induced Conditioned Place Preference. Front. Mol. Neurosci. 10, 313. 10.3389/fnmol.2017.00313 29051727 PMC5633679

[B39] Kazemi T. Huang S. Avci N. G. Waits C. M. K. Akay Y. M. Akay M. (2020). Investigating the Influence of Perinatal Nicotine and Alcohol Exposure on the Genetic Profiles of Dopaminergic Neurons in the VTA Using miRNA-mRNA Analysis. Sci. Rep. 10 (1), 15016. 10.1038/s41598-020-71875-1 32929144 PMC7490691

[B40] Keller R. F. Dragomir A. Yantao F. Akay Y. M. Akay M. (2018). Investigating the Genetic Profile of Dopaminergic Neurons in the VTA in Response to Perinatal Nicotine Exposure Using mRNA-miRNA Analyses. Sci. Rep. 8 (1), 13769. 10.1038/s41598-018-31882-9 30213973 PMC6137108

[B41] Kim B. Yun J. Park B. (2020). Methamphetamine-Induced Neuronal Damage: Neurotoxicity and Neuroinflammation. Biomol. Ther. (Seoul) 28 (5), 381–388. 10.4062/biomolther.2020.044 32668144 PMC7457172

[B42] Kurinna S. Werner S. (2015). NRF2 and microRNAs: New but Awaited Relations. Biochem. Soc. Trans. 43 (4), 595–601. 10.1042/BST20140317 26551699

[B43] Lee S. W. L. Paoletti C. Campisi M. Osaki T. Adriani G. Kamm R. D. (2019). MicroRNA Delivery through Nanoparticles. J. Control. Release 313, 80–95. 10.1016/j.jconrel.2019.10.007 31622695 PMC6900258

[B44] Leggio L. Vivarelli S. L'Episcopo F. Tirolo C. Caniglia S. Testa N. (2017). microRNAs in Parkinson's Disease: From Pathogenesis to Novel Diagnostic and Therapeutic Approaches. Int. J. Mol. Sci. 18 (12). 10.3390/ijms18122698 PMC5751299 29236052

[B45] Li F. Li D. Tang S. Liu J. Yan J. Chen H. (2021a). Quercetin Protects H9c2 Cardiomyocytes against Oxygen-Glucose Deprivation/Reoxygenation-Induced Oxidative Stress and Mitochondrial Apoptosis by Regulating the ERK1/2/DRP1 Signaling Pathway. Evid. Based Complement. Alternat Med. 2021, 7522175. 10.1155/2021/7522175 34457029 PMC8390138

[B46] Li F. Liu J. Tang S. Yan J. Chen H. Li D. (2021b). Quercetin Regulates Inflammation, Oxidative Stress, Apoptosis, and Mitochondrial Structure and Function in H9C2 Cells by Promoting PVT1 Expression. Acta Histochem. 123 (8), 151819. 10.1016/j.acthis.2021.151819 34844154

[B47] Li H. Li C. Zhou Y. Luo C. Ou J. Li J. (2018). Expression of microRNAs in the Serum Exosomes of Methamphetamine-dependent Rats vs. Ketamine-dependent Rats. Exp. Ther. Med. 15 (4), 3369–3375. 10.3892/etm.2018.5814 29545857 PMC5840958

[B48] Li J. Zhu L. Su H. Liu D. Yan Z. Ni T. (2021c). Regulation of miR-128 in the Nucleus Accumbens Affects Methamphetamine-Induced Behavioral Sensitization by Modulating Proteins Involved in Neuroplasticity. Addict. Biol. 26 (1), e12881. 10.1111/adb.12881 32058631

[B49] Li M. D. van der Vaart A. D. (2011). MicroRNAs in Addiction: Adaptation's Middlemen? Mol. Psychiatry 16 (12), 1159–1168. 10.1038/mp.2011.58 21606928 PMC4251867

[B50] Liao K. Niu F. Hu G. Yang L. Dallon B. Villarreal D. (2020). Morphine-mediated Release of miR-138 in Astrocyte-Derived Extracellular Vesicles Promotes Microglial Activation. J. Extracell Vesicles 10 (1), e12027. 10.1002/jev2.12027 33304479 PMC7710131

[B51] Liao L. S. Lu S. Yan W. T. Wang S. C. Guo L. M. Yang Y. D. (2021). The Role of HSP90α in Methamphetamine/Hyperthermia-Induced Necroptosis in Rat Striatal Neurons. Front. Pharmacol. 12, 716394. 10.3389/fphar.2021.716394 34349659 PMC8326403

[B52] Lipton D. M. Gonzales B. J. Citri A. (2019). Dorsal Striatal Circuits for Habits, Compulsions and Addictions. Front. Syst. Neurosci. 13, 28. 10.3389/fnsys.2019.00028 31379523 PMC6657020

[B53] Liu D. Liang M. Zhu L. Zhou T. T. Wang Y. Wang R. (2021). Potential Ago2/miR-3068-5p Cascades in the Nucleus Accumbens Contribute to Methamphetamine-Induced Locomotor Sensitization of Mice. Front. Pharmacol. 12, 708034. 10.3389/fphar.2021.708034 34483916 PMC8414410

[B54] Liu D. Zhu L. Ni T. Guan F. L. Chen Y. J. Ma D. L. (2019). Ago2 and Dicer1 Are Involved in METH-Induced Locomotor Sensitization in Mice via Biogenesis of miRNA. Addict. Biol. 24 (3), 498–508. 10.1111/adb.12616 29516602

[B55] Lu T. X. Rothenberg M. E. (2018). MicroRNA. J. Allergy Clin. Immunol. 141 (4), 1202–1207. 10.1016/j.jaci.2017.08.034 29074454 PMC5889965

[B56] Ma F. Zhang X. Yin K. J. (2020). MicroRNAs in central Nervous System Diseases: A Prospective Role in Regulating Blood-Brain Barrier Integrity. Exp. Neurol. 323, 113094. 10.1016/j.expneurol.2019.113094 31676317 PMC7304262

[B57] Marangon D. Raffaele S. Fumagalli M. Lecca D. (2019). MicroRNAs Change the Games in central Nervous System Pharmacology. Biochem. Pharmacol. 168, 162–172. 10.1016/j.bcp.2019.06.019 31251938

[B58] Matsumoto R. R. Seminerio M. J. Turner R. C. Robson M. J. Nguyen L. Miller D. B. (2014). Methamphetamine-induced Toxicity: an Updated Review on Issues Related to Hyperthermia. Pharmacol. Ther. 144 (1), 28–40. 10.1016/j.pharmthera.2014.05.001 24836729 PMC4700537

[B59] McDonnell-Dowling K. Kelly J. P. (2017). The Role of Oxidative Stress in Methamphetamine-Induced Toxicity and Sources of Variation in the Design of Animal Studies. Curr. Neuropharmacol 15 (2), 300–314. 10.2174/1570159x14666160428110329 27121285 PMC5412700

[B60] Mellios N. Huang H. S. Grigorenko A. Rogaev E. Akbarian S. (2008). A Set of Differentially Expressed miRNAs, Including miR-30a-5p, Act as post-transcriptional Inhibitors of BDNF in Prefrontal Cortex. Hum. Mol. Genet. 17 (19), 3030–3042. 10.1093/hmg/ddn201 18632683 PMC2722882

[B61] Meng X. Zhang C. Guo Y. Han Y. Wang C. Chu H. (2020). TBHQ Attenuates Neurotoxicity Induced by Methamphetamine in the VTA through the Nrf2/HO-1 and PI3K/AKT Signaling Pathways. Oxid Med. Cel Longev 2020, 8787156. 10.1155/2020/8787156 PMC7174937 32351675

[B62] Meng Y. Zhang Y. Tregoubov V. Falls D. L. Jia Z. (2003). Regulation of Spine Morphology and Synaptic Function by LIMK and the Actin Cytoskeleton. Rev. Neurosci. 14 (3), 233–240. 10.1515/revneuro.2003.14.3.233 14513866

[B63] Mizoguchi H. Yamada K. (2019). Methamphetamine Use Causes Cognitive Impairment and Altered Decision-Making. Neurochem. Int. 124, 106–113. 10.1016/j.neuint.2018.12.019 30611760

[B64] Monica D. Prakash K. T. Antonipillai J. Stojanovska L. Nurgali1 K. Apostolopoulos V. (2017). Methamphetamine: Effects on the Brain, Gut and Immune System. Pharmacol. Res. 120, 60–67. 28302577 10.1016/j.phrs.2017.03.009

[B65] Moratalla R. Khairnar A. Simola N. Granado N. García-Montes J. R. Porceddu P. F. (2017). Amphetamine-related Drugs Neurotoxicity in Humans and in Experimental Animals: Main Mechanisms. Prog. Neurobiol. 155, 149–170. 10.1016/j.pneurobio.2015.09.011 26455459

[B66] Moszczynska A. Callan S. P. (2017). Molecular, Behavioral, and Physiological Consequences of Methamphetamine Neurotoxicity: Implications for Treatment. J. Pharmacol. Exp. Ther. 362 (3), 474–488. 10.1124/jpet.116.238501 28630283 PMC11047030

[B67] Mulligan M. K. Dubose C. Yue J. Miles M. F. Lu L. Hamre K. M. (2013). Expression, Covariation, and Genetic Regulation of miRNA Biogenesis Genes in Brain Supports Their Role in Addiction, Psychiatric Disorders, and Disease. Front. Genet. 4, 126. 10.3389/fgene.2013.00126 23847651 PMC3701868

[B68] Ni T. Li Y. Wang R. Hu T. Guan F. Zhu L. (2019). The Potential Involvement of miR-204-3p-Axon Guidance Network in Methamphetamine-Induced Locomotor Sensitization of Mice. Neurosci. Lett. 707, 134303. 10.1016/j.neulet.2019.134303 31153969

[B69] Nunomura A. Perry G. (2020). RNA and Oxidative Stress in Alzheimer's Disease: Focus on microRNAs. Oxid Med. Cel Longev 2020, 2638130. 10.1155/2020/2638130 PMC7721489 33312335

[B70] Oshima S. Asai S. Seki N. Minemura C. Kinoshita T. Goto Y. (2021). Identification of Tumor Suppressive Genes Regulated by miR-31-5p and miR-31-3p in Head and Neck Squamous Cell Carcinoma. Ijms 22 (12), 6199. 10.3390/ijms22126199 34201353 PMC8227492

[B71] Park J. H. Seo Y. H. Jang J. H. Jeong C. H. Lee S. Park B. (2017). Asiatic Acid Attenuates Methamphetamine-Induced Neuroinflammation and Neurotoxicity through Blocking of NF-kB/STAT3/ERK and Mitochondria-Mediated Apoptosis Pathway. J. Neuroinflammation 14 (1), 240. 10.1186/s12974-017-1009-0 29228978 PMC5725763

[B72] Periyasamy P. Liao K. Kook Y. H. Niu F. Callen S. E. Guo M. L. (2018). Cocaine-Mediated Downregulation of miR-124 Activates Microglia by Targeting KLF4 and TLR4 Signaling. Mol. Neurobiol. 55 (4), 3196–3210. 10.1007/s12035-017-0584-5 28478506 PMC5673594

[B73] Preston A. R. Eichenbaum H. (2013). Interplay of hippocampus and Prefrontal Cortex in Memory. Curr. Biol. 23 (17), R764–R773. 10.1016/j.cub.2013.05.041 24028960 PMC3789138

[B74] Puerta-Gil P. García-Baquero R. Jia A. Y. Ocaña S. Alvarez-Múgica M. Alvarez-Ossorio J. L. (2012). miR-143, miR-222, and miR-452 Are Useful as Tumor Stratification and Noninvasive Diagnostic Biomarkers for Bladder Cancer. Am. J. Pathol. 180 (5), 1808–1815. 10.1016/j.ajpath.2012.01.034 22426337

[B75] Qian H. Shang Q. Liang M. Gao B. Xiao J. Wang J. (2021). MicroRNA-31-3p/RhoA Signaling in the Dorsal hippocampus Modulates Methamphetamine-Induced Conditioned Place Preference in Mice. Psychopharmacology (Berl) 238 (11), 3207–3219. 10.1007/s00213-021-05936-2 34313802

[B76] Ramkissoon A. Wells P. G. (2015). Methamphetamine Oxidative Stress, Neurotoxicity, and Functional Deficits Are Modulated by Nuclear Factor-E2-Related Factor 2. Free Radic. Biol. Med. 89, 358–368. 10.1016/j.freeradbiomed.2015.07.157 26427884

[B77] Saliminejad K. Khorram Khorshid H. R. Soleymani Fard S. Ghaffari S. H. (2019). An Overview of microRNAs: Biology, Functions, Therapeutics, and Analysis Methods. J. Cel Physiol 234 (5), 5451–5465. 10.1002/jcp.27486 30471116

[B78] Sambo D. O. Lebowitz J. J. Khoshbouei H. (2018). The Sigma-1 Receptor as a Regulator of Dopamine Neurotransmission: A Potential Therapeutic Target for Methamphetamine Addiction. Pharmacol. Ther. 186, 152–167. 10.1016/j.pharmthera.2018.01.009 29360540 PMC5962385

[B79] Sandau U. S. Duggan E. Shi X. Smith S. J. Huckans M. Schutzer W. E. (2020). Methamphetamine Use Alters Human Plasma Extracellular Vesicles and Their microRNA Cargo: An Exploratory Study. J. Extracell Vesicles 10 (1), e12028. 10.1002/jev2.12028 33613872 PMC7890470

[B80] Sengupta J. N. Pochiraju S. Pochiraju S. Kannampalli P. Bruckert M. Addya S. (2013). MicroRNA-mediated GABA Aα-1 Receptor Subunit Down-Regulation in Adult Spinal Cord Following Neonatal Cystitis-Induced Chronic Visceral Pain in Rats. Pain 154 (1), 59–70. 10.1016/j.pain.2012.09.002 23273104 PMC3535325

[B81] Shaerzadeh F. Streit W. J. Heysieattalab S. Khoshbouei H. (2018). Methamphetamine Neurotoxicity, Microglia, and Neuroinflammation. J. Neuroinflammation 15 (1), 341. 10.1186/s12974-018-1385-0 30541633 PMC6292109

[B82] Shi J. J. Cao D. N. Liu H. F. Wang Z. Y. Lu G. Y. Wu N. (2019). Dorsolateral Striatal miR-134 Modulates Excessive Methamphetamine Intake in Self-Administering Rats. Metab. Brain Dis. 34 (4), 1029–1041. 10.1007/s11011-019-00430-3 31152340

[B83] Shin E. J. Tran H. Q. Nguyen P. T. Jeong J. H. Nah S. Y. Jang C. G. (2018). Role of Mitochondria in Methamphetamine-Induced Dopaminergic Neurotoxicity: Involvement in Oxidative Stress, Neuroinflammation, and Pro-apoptosis-A Review. Neurochem. Res. 43 (1), 66–78. 10.1007/s11064-017-2318-5 28589520

[B84] Sim M. S. Soga T. Pandy V. Wu Y. S. Parhar I. S. Mohamed Z. (2017). MicroRNA Expression Signature of Methamphetamine Use and Addiction in the Rat Nucleus Accumbens. Metab. Brain Dis. 32 (6), 1767–1783. 10.1007/s11011-017-0061-x 28681200

[B85] Singh R. Saini N. (2012). Downregulation of BCL2 by miRNAs Augments Drug-Induced Apoptosis-Aa Combined Computational and Experimental Approach. J. Cel Sci 125 (Pt 6), 1568–1578. 10.1242/jcs.095976 22328513

[B86] Srivastava N. Manvati S. Srivastava A. Pal R. Kalaiarasan P. Chattopadhyay S. (2011). miR-24-2 Controls H2AFX Expression Regardless of Gene Copy Number Alteration and Induces Apoptosis by Targeting Antiapoptotic Gene BCL-2: a Potential for Therapeutic Intervention. Breast Cancer Res. 13 (2), R39. 10.1186/bcr2861 21463514 PMC3219202

[B87] Su H. Zhu L. Li J. Wang R. Liu D. Han W. (2019). Regulation of microRNA-29c in the Nucleus Accumbens Modulates Methamphetamine -induced Locomotor Sensitization in Mice. Neuropharmacology 148, 160–168. 10.1016/j.neuropharm.2019.01.007 30639389

[B88] Tehrani A. M. Boroujeni M. E. Aliaghaei A. Feizi M. A. H. Safaralizadeh R. (2019). Methamphetamine Induces Neurotoxicity-Associated Pathways and Stereological Changes in Prefrontal Cortex. Neurosci. Lett. 712, 134478. 10.1016/j.neulet.2019.134478 31491463

[B89] van der Pol A. van Gilst W. H. Voors A. A. van der Meer P. (2019). Treating Oxidative Stress in Heart Failure: Past, Present and Future. Eur. J. Heart Fail. 21 (4), 425–435. 10.1002/ejhf.1320 30338885 PMC6607515

[B90] Volkow N. D. Michaelides M. Baler R. (2019). The Neuroscience of Drug Reward and Addiction. Physiol. Rev. 99 (4), 2115–2140. 10.1152/physrev.00014.2018 31507244 PMC6890985

[B91] Volkow N. D. Morales M. (2015). The Brain on Drugs: From Reward to Addiction. Cell 162 (4), 712–725. 10.1016/j.cell.2015.07.046 26276628

[B92] Wang Y. Wei T. Zhao W. Ren Z. Wang Y. Zhou Y. (2021). MicroRNA-181a Is Involved in Methamphetamine Addiction through the ERAD Pathway. Front. Mol. Neurosci. 14, 667725. 10.3389/fnmol.2021.667725 34025353 PMC8137846

[B93] Xia X. Wang Y. Huang Y. Zhang H. Lu H. Zheng J. C. (2019). Exosomal miRNAs in central Nervous System Diseases: Biomarkers, Pathological Mediators, Protective Factors and Therapeutic Agents. Prog. Neurobiol. 183, 101694. 10.1016/j.pneurobio.2019.101694 31542363 PMC7323939

[B94] Yan P. Xu D. Ji Y. Yin F. Cui J. Su R. (2019). LiCl Pretreatment Ameliorates Adolescent Methamphetamine Exposure-Induced Long-Term Alterations in Behavior and Hippocampal Ultrastructure in Adulthood in Mice. Int. J. Neuropsychopharmacol. 22 (4), 303–316. 10.1093/ijnp/pyz001 30649326 PMC6441133

[B95] Yang J. Li L. Hong S. Zhang D. Zhou Y. (2020). Methamphetamine Leads to the Alterations of microRNA Profiles in the Nucleus Accumbens of Rats. Pharm. Biol. 58 (1), 797–805. 10.1080/13880209.2020.1803366 32893733 PMC8641683

[B96] Yang X. Wang Y. Li Q. Zhong Y. Chen L. Du Y. (2018). The Main Molecular Mechanisms Underlying Methamphetamine- Induced Neurotoxicity and Implications for Pharmacological Treatment. Front. Mol. Neurosci. 11, 186. 10.3389/fnmol.2018.00186 29915529 PMC5994595

[B97] Yu G. Song Y. Xie C. Tao L. Wan F. Jiang L. (2019). MiR-142a-3p and miR-155-5p Reduce Methamphetamine-Induced Inflammation: Role of the Target Protein Peli1. Toxicol. Appl. Pharmacol. 370, 145–153. 10.1016/j.taap.2019.03.019 30914375

[B98] Yu S. Zhu L. Shen Q. Bai X. Di X. (2015). Recent Advances in Methamphetamine Neurotoxicity Mechanisms and its Molecular Pathophysiology. Behav. Neurol. 2015, 103969. 10.1155/2015/103969 25861156 PMC4377385

[B99] Zhang F. Huang S. Bu H. Zhou Y. Chen L. Kang Z. (2021). Disrupting Reconsolidation by Systemic Inhibition of mTOR Kinase via Rapamycin Reduces Cocaine-Seeking Behavior. Front. Pharmacol. 12, 652865. 10.3389/fphar.2021.652865 33897438 PMC8064688

[B100] Zhang Y. Wang Z. Gemeinhart R. A. (2013). Progress in microRNA Delivery. J. Control. Release 172 (3), 962–974. 10.1016/j.jconrel.2013.09.015 24075926 PMC3891846

[B101] Zhao M. W. Yang P. Zhao L. L. (2019). Chlorpyrifos Activates Cell Pyroptosis and Increases Susceptibility on Oxidative Stress-Induced Toxicity by miR-181/SIRT1/PGC-1α/Nrf2 Signaling Pathway in Human Neuroblastoma SH-Sy5y Cells: Implication for Association between Chlorpyrifos and Parkinson's Disease. Environ. Toxicol. 34 (6), 699–707. 10.1002/tox.22736 30835941

[B102] Zhou Q. Anderson C. Zhang H. Li X. Inglis F. Jayagopal A. (2014). Repression of Choroidal Neovascularization through Actin Cytoskeleton Pathways by microRNA-24. Mol. Ther. 22 (2), 378–389. 10.1038/mt.2013.243 24297048 PMC3916039

[B103] Zhou Y. Xiao S. Li C. Chen Z. Zhu C. Zhou Q. (2021). Extracellular Vesicle-Encapsulated miR-183-5p from Rhynchophylline-Treated H9c2 Cells Protect against Methamphetamine-Induced Dependence in Mouse Brain by Targeting NRG1. Evid. Based Complement. Alternat Med. 2021, 2136076. 10.1155/2021/2136076 34484386 PMC8416368

